# Cerebro-Spinal Fever in the Newer Light

**Published:** 1917-03-10

**Authors:** 


### CEREBRO-SPINAL FEVER IN THE NEWER LIGHT.

### The Community versus the Microbe.

AN epidemic disease in the light of modern medical knowledge involves the suggestion that the essential cause of the disease is a living organism. Even long before the days of bacteriological demonstration there was something more than a suspicion that an illness which spread more or less rapidly and more or less generally through the members of a community must depend upon a *contagium vivum*. Nothing less than an agent which possessed the power of self-multiplication seemed capable of explaining the extension of the disease from person to person, from house to house, from town to town. Towards the same conclusion pointed the fact that throughout the area or areas affected an epidemic always "bred true." In each instance there was scarlet fever throughout, or measles throughout, or smallpox throughout; that is, the later cases always conformed to the one or more recognised as the starting-point of the outbreak. This experience carried the presumption, not only that epidemic disease depended upon living organisms, but also that each variety of epidemic disease depended on a specific and individual form of organism. In time came an actual demonstration of many of these organisms, so that what had hitherto been a suspicion or speculation was established as scientific certainty. So convincing is the evidence on this point that even in epidemic diseases where no causal organism has been discovered the existence of such an organism is almost universally accepted; what is lacking, such is the modern judgment, is not the organism but the means to isolate and identify it. When this position is recognised it follows that the existence of an epidemic disease imposes upon the medical profession two different though related ambitions. One of these concerns the individual patient; the other, the community. The needs of the patient are that his symptoms shall be correctly interpreted, and that all possible measures shall be adopted to bring him safely through his illness. The community also is interested in correct diagnosis, seeing that any sufferer is a possible source of danger to his fellows, and this more especially if the true nature of the case is unrecognised. But there is a still wider claim—namely, that the medical profession shall discover the channel or method by which the disease is spread and the means by which such spread may be avoided. While the individual patient asks for cure or successful treatment, the demand of the community is for prevention. And for the reasons given above, successful prevention must mean (1) the identification of the organism which is the essential cause of the disease, (2) a knowledge of the method by which this organism gains access to the bodies of human beings, and (3) the creation of agencies which will either destroy the organism or will restrain its activities. Such, in general terms, is the campaign imposed upon the medical profession by the existence of epidemic disease—to discover the specific cause, to treat the individual patient, to protect the unaffected members of the community.

The principles here stated have received an effective illustration in the latest Report of the Medical Research Committee dealing with the subject of cerebro-spinal fever. The outbreaks of this disease which have occurred during the last two years in certain military camps have given to the subject an urgency and importance which, at least in this country, it had not previously possessed. Earlier epidemics of the disease in the United Kingdom had been on but a limited scale, and a special responsibility was naturally felt when young men training for the fighting services were attacked. Hence both the military authorities and public opinion demanded that every effort should be made to check the outbreak and so both to protect the lives of our young soldiers and to avert the danger from the civil population. Fortunately, an instrument suitable for this purpose existed in the shape of the Medical Research Committee, and the latest Report of the Committee shows what has been accomplished as a result of a scientific study of the various problems involved, and of the application to these problems of organised observation and research. The whole Report demands careful study and is full of interest. Here we may briefly indicate some of the practical results in so far as they bear on the diagnosis, treatment, and prevention of the disease.

On the symptomatic display of the disease there is little new to record. In view of the recent activity of influenza it may, however, be well to remark that in not a few cases of cerebro-spinal fever there may be for some days little beyond general febrile disturbance and headache. Hence it is easy, especially when influenza is prevalent, to regard as influenza a case which needs a much more serious diagnosis. The picture of cerebro-spinal fever, in short, is in clinical practice very often extremely incomplete as compared with the systematic descriptions of the disease, and particularly it may be noted that in many, and even in the majority of cases, no skin eruption appears, so that the term "spotted fever" is far from being a happy one. This want of precision in the clinical evidence makes it all the more important that the practitioner shall be ready to avail himself of the help to be obtained by laboratory tests. The value of an examination of the cerebro-spinal fluid can hardly be exaggerated, and in no case in which there is the slightest ground for suspicion should it be omitted. Stress is now laid also on the discovery of meningococci in the naso-pharynx, as this fact can be determined by a properly applied swab. And, again, there is some value in an agglutination test applied to the blood, and similar in principle to the Widal test for typhoid fever. The refined processes by which the specific meningococci are recognised as distinct from other cocci more or less closely resembling them involve special methods which cannot be developed outside a well-equipped laboratory. They must be left in the hands of the expert bacteriologist, but the responsibility of recognising when, with a view to diagnosis, such investigations are required rests with the medical practitioner, and he must be prepared for it.

In reference to the active treatment of cerebro-spinal fever, there has been much discussion during the last year or two on the value of an anti-meningococcic serum injected into the spinal canal after the previous withdrawal of a corresponding volume of the cerebro-spinal fluid. Some writers have gone so far as to hold that the sole beneficial influence in this procedure is the removal of the fluid by the lumbar puncture, and hence, while urging the frequent repetition of this puncture, they have opposed the subsequent introduction of a serum. This is the course advised by Dr. Foster and Dr. Gaskell in the extremely valuable and informing book which they published last year (Cambridge University Press). Others have reported good results from the injection of serum, and have explained failures as due to a non-correspondence between the particular type or strain of the organism present and the variety of serum employed. It is allowed that there are various types of the organism, and we have in the Report here discussed an account of the work undertaken with a view to isolate and identify these; there is, too, evidence to show that as a matter of fact a serum which is efficient against one type is useless in the case of others. As a practical outcome there has been produced a serum prepared from more than one type of the organism—the Millbank-Lister Institute serum—and this, we are told, has yielded exceedingly satisfactory results. Early administration and frequent administration are points of capital importance. Not less than three doses, each of 30 c.c., are given at intervals of twenty-four hours, and this, be it observed, however favourable may be the course of the case. There is in this connection a recommendation which ought to be carefully noted. It is that the lumbar puncture should be practised when the patient is under the influence of a general anæsthetic. One reason for this is that the anæsthetic causes a rise in the cerebro-spinal fluid pressure, and hence a free flow of the fluid—even to 70 to 100 c.c.—is easily obtained. It is generally recognised that the volume of serum injected must never exceed the volume of cerebro-spinal fluid withdrawn, and not infrequently hitherto there has been difficulty in getting such an amount of fluid as would justify the administration of the serum in full doses. The observation just recorded removes this difficulty, while at the same time its adoption in practice facilitates the task of the operator and spares pain to the patient. Altogether the effect of the Report is in favour of treatment by a properly prepared serum, and it is to be noted that apart from some instances of "serum rash" no unfavourable symptoms followed the use of such a serum. Finally, under the section on treatment, it is recorded that in cases which have been slow to improve a sensitised vaccine prepared from the patient's own coccus has been used with advantage.

Special attention is demanded by the observations and experiments made with a view to the prevention of cerebro-spinal fever. It has been known for some time that the dangerous person from the point of view of the community is less the patient who is the victim of meningitis than one who, while apparently in perfect health, harbours the meningococcus in his naso-pharynx. It is these "carriers" who are mainly responsible for the spread of the disease. By spitting, hawking, and coughing they discharge the germ into the air, and it thus gains access to the respiratory tracts of their friends and neighbours. At the same time it has now been established that the cerebro-spinal fever patient is himself a "carrier" in the sense that the meningococcus is present in his naso-pharynx. Hence in a hospital ward it is found that neighbouring patients, nurses, and so on become "carriers." There thus arises the obvious suggestion that all persons who have been more or less in contact with a cerebro-spinal fever patient shall have a swab taken from the throat, and that if this shows the presence of meningococci any such person shall be isolated until the throat is free from these. It has therefore become the practice in military hospitals to swab all "contacts" and to isolate those who are found to be "carriers," and this in individual instances may mean a large number of observations, for by the time the diagnosis is secured "carriers" have probably been created, and these in turn have had their series of "contacts." In a word, starting from the patient there are "direct contacts," some of whom may prove to be "carriers," and these in turn have their "contacts," that is "indirect contacts" as regards the original patient. With men under military discipline the task is a comparatively simple one, but it is easy to see how difficult such a series of examinations would be in civil practice. All the more reason, therefore, why due appreciation should be extended to the efforts of the Medical Research Committee and their experts.

Some of the newer knowledge on the subject of "carriers" may be briefly stated. First it has been established that not all the cocci found in the naso-pharynx are capable of causing cerebro-spinal fever, and by certain tests it is now possible to distinguish the harmless varieties from the dangerous ones. Hence a certain number of persons who would formerly have been condemned as "carriers" and put in isolation are able to be discharged. Secondly, it has been shown that of the dangerous cocci there are several varieties—four at least—capable of producing cerebro-spinal fever, and that in a given patient the same type of organism which is present in his naso-pharynx is present also in his cerebro-spinal fluid. Further, there is evidence that when serum is used for curative purposes it must correspond to the particular strain of coccus which has attacked the patient if good results are to be obtained. Hence the problem of the "carrier" is a very complex one, and the advance to the present position has meant a highly organised and persevering plan of campaign. The finished work may be stated in a few words, but the technical studies on which the accomplishment rests have involved much patience and a high measure of ingenuity. One further point on the subject of "carriers." Naturally it is asked whether, when the "carrier" is caught, something cannot be done to get rid of the organisms which he is sheltering in his nasopharynx. Observations show that in time—days or weeks—he becomes harmless in this respect. The organisms tend to die out under sound hygienic conditions. To hasten this process is obviously a highly desirable thing to do, and numerous antiseptic applications in various forms have been attempted. So far no completely satisfactory result can be claimed, but some encouraging observations are recorded with the inhalation of an atmosphere containing chloramine. The prompt and certain conversion of the "carrier" from a dangerous into a harmless individual remains for achievement, and the omens are that it will not long be delayed.

